# *Wolbachia* restricts insect-specific flavivirus infection in *Aedes aegypti* cells

**DOI:** 10.1099/jgv.0.000617

**Published:** 2016-11-10

**Authors:** Esther Schnettler, Vatipally B. Sreenu, Timothy Mottram, Melanie McFarlane

**Affiliations:** ^1^​ MRC–University of Glasgow Centre for Virus Research, Glasgow G61 1QH, Scotland, UK

**Keywords:** *Wolbachia*, insect-specific virus, CFAV, PCLV, *A. aegypti*

## Abstract

Mosquito-borne viruses are known to cause disease in humans and livestock and are often difficult to control due to the lack of specific antivirals and vaccines. The *Wolbachia* endosymbiont has been widely studied for its ability to restrict positive-strand RNA virus infection in mosquitoes, although little is known about the precise antiviral mechanism. In recent years, a variety of insect-specific viruses have been discovered in mosquitoes and an interaction with mosquito-borne viruses has been reported for some of them; however, nothing is known about the effect of *Wolbachia* on insect-specific virus infection in mosquitoes. Here, we show that transinfection of the *Drosophila*-derived *w*MelPop *Wolbachia* strain into *Aedes aegypti*-derived cells resulted in inhibition and even clearance of the persistent cell-fusing agent flavivirus infection in these cells. This broadens the antiviral activity of *Wolbachia* from acute infections to persistent infections and from arboviruses to mosquito-specific viruses. In contrast, no effect on the Phasi Charoen-like bunyavirus persistent infection in these cells was observed, suggesting a difference in *Wolbachia* inhibition between positive- and negative-strand RNA viruses.

## Introduction

Arboviruses are comprised of human and animal pathogens that are transmitted via blood-feeding arthropod vectors, including mosquitoes. Due to the lack of efficient antivirals and vaccines against most of these viruses, vector control is an important intervention strategy to reduce the impact of these viruses on human and animal health (Kean *et al.*, 2015; Weaver & Reisen, 2010). In recent years, the use of the endosymbiotic intracellular bacterium, *Wolbachia*, has been a well-studied approach to control arbovirus transmission by mosquitoes and in particular by *Aedes aegypti* (Iturbe-Ormaetxe *et al.*, 2011; Rainey *et al.*, 2014). *Wolbachia* was first shown to confer resistance to RNA viruses in *Drosophila*-virus systems (Hedges *et al.*, 2008; Teixeira *et al.*, 2008). Later, transinfection of *Drosophila*-derived *Wolbachia* into *A. aegypti* (which is not known to naturally harbour these endosymbionts) or its derived cell lines resulted in resistance to the important mosquito-borne dengue (DENV) and chikungunya viruses (Moreira *et al.*, 2009; Walker *et al.*, 2011). This has resulted in successful field trials of *A. aegypti* transinfected with *Wolbachia*, proving its ability to reduce DENV transmission in natural settings (Frentiu *et al.*, 2014). Moreover, *Wolbachia* can be stably maintained in nature, as crosses between non-infected females and infected males do not result in any offspring (Hoffmann *et al.*, 2011, 2014). This unique feature is called cytoplasmic incapability (McMeniman *et al.*, 2009) and gives a reproductive advantage to infected female mosquitoes, resulting in the spread of *Wolbachia* through the mosquito population (Sinkins, 2004).

The mechanism(s) of virus inhibition through *Wolbachia* is not known. Inhibition has been linked to *Wolbachia* density, with the resistant phenotype observed only with *Wolbachia* strains producing high concentrations of bacteria in infected cells (Osborne *et al.*, 2009, 2012). Recent findings show the ability of *Wolbachia* to interfere with early events in virus replication, suggesting an intrinsic mechanism for viral resistance (Rainey *et al.*, 2016).

It should be noted that *Wolbachia*-mediated virus resistance has only been reported for positive-stranded RNA viruses and no resistance has yet been reported for negative-stranded RNA viruses (Rainey *et al.*, 2014), which include a variety of important mosquito-borne viruses such as *Rift Valley fever virus* (*Bunyaviridae*).

Further to arboviruses, mosquitoes have also been shown to be infected with additional viruses, called insect-specific viruses (ISVs) as they replicate exclusively in insect cells. The list of ISVs is steadily increasing through novel identification methods, including next-generation sequencing. ISVs belong to different virus families/genera, including the *Bunyaviridae* and *Flaviviridae* families, which also include important arboviruses. ISVs belonging to the *Flavivirus* genus share sequence similarities with their arbovirus counterparts, but cluster as a single defined group suggesting independent evolution. In contrast, ISVs belonging to the *Bunyaviridae* cluster into several defined groups across the virus family (Bolling *et al.*, 2015; Marklewitz *et al.*, 2015). ISV infections, at least in cell culture, normally result in initial cytopathic effect, followed by progression into a persistent, non-cytopathic infection (Bolling *et al.*, 2015; Marklewitz *et al.*, 2015).

The increasing numbers of ISVs identified in mosquitoes and derived cells suggest that a large number of mosquitoes in the wild are naturally infected with ISVs and that vertical transmission is the main infection and maintenance route. Thereby one can expect that mosquitoes in the wild can be infected by several viruses, including ISVs and/or arboviruses. Moreover, the interaction between ISV and arbovirus infections (either co-infected or sequentially infected) results in either inhibition or increased replication/infection of one of the viruses (Kean *et al.*, 2015). It is suggested that such interactions could partly define vector competence of a mosquito in the wild to a given arbovirus.

No information is available at the moment about the interaction of *Wolbachia* with these ISVs or what effect *Wolbachia* transinfection could have on mosquitoes already persistently infected with RNA viruses. The inhibitory effect of *Wolbachia* on RNA viruses has only been investigated in light of an acute virus infection following a persistent *Wolbachia* transinfection (Rainey *et al.*, 2014).

In order to address these questions and to understand if *Wolbachia* interacts with acute or persistent infections of ISVs, we have used the *A. aegypti*-derived Aag2 cell line previously transinfected with the *Drosophila*-derived *Wolbachia* strain *w*MelPop (known to grow to high titres and mediate DENV resistance) (Hedges *et al.*, 2008; Teixeira *et al.*, 2008) to investigate the effect of *Wolbachia* on two ISVs, known to be present in Aag2 cells and belonging to different families: positive-strand RNA cell-fusing agent virus (CFAV, *Flaviviridae*) (Scott *et al.*, 2010) and the negative-strand RNA Phasi Charoen-like bunyavirus (PCLV, *Bunyaviridae*) (Maringer *et al.*, 2015). Our results show that *Wolbachia* can refer resistance to CFAV infection independently of the time of *Wolbachia* transinfection. In contrast, no viral inhibition by *Wolbachia* was observed for PCLV in these experiments.

## Results

### Effect of *Wolbachia* on small RNA production in Aag2 cells

**Fig. 1.**
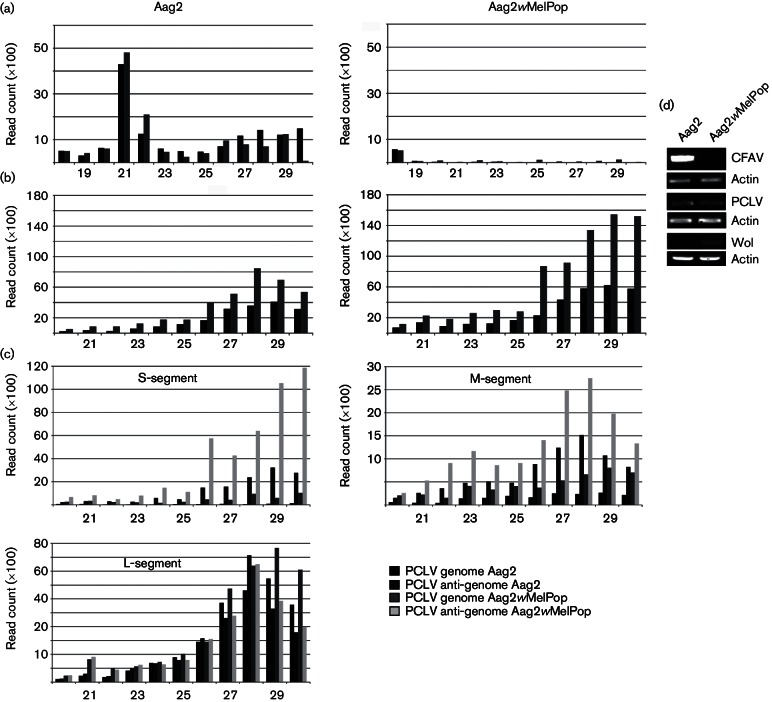
Presence or absence of CFAV, PCLV and *w*MelPop in Aag2 and Aag2*w*MelPop cells. Size distribution of small RNA molecules mapping to the CFAV (a) or PCLV (b) genome (black)/anti-genome (grey) in *A. aegypti*-derived Aag2 or *w*MelPop-transinfected Aag2 cells. (c) Size distribution of small RNA molecules mapping to the different segments of PCLV (S, M and L) genome/anti-genome in *A. aegypti*-derived Aag2 or *w*MelPop-transinfected Aag2 cells. (d) Detection of CFAV or PCLV in Aag2 and Aag2*w*MelPop cells by RT-PCR. Actin was used as loading control.

Aag2 cells can be stably transinduced with the *w*MelP strain of *Drosophila*, resulting in a reduction of small RNAs in the cytoplasm due to inhibition of small RNA transport from the nucleus to the cytoplasm (Mayoral *et al.*, 2014). Aag2 cells are known to be persistently infected with the insect-specific flavivirus, CFAV and as result produce CFAV-specific small RNAs (Scott *et al.*, 2010). Recently it has also been reported that Aag2 cells produce transcripts and proteins from another ISV, PCLV (suggesting a persistent infection) (Maringer *et al.*, 2015). However, it is not yet known if this is due to an active virus infection. This virus has also been recently discovered in wild mosquitoes in Brazil (Aguiar *et al.*, 2015). Therefore, we re-analysed the previously reported small RNA data of Aag2 and Aag2*w*MelPop cells (Mayoral *et al.*, 2014) and mapped them to CFAV or PCLV. Almost no small RNA reads were detected in Aag2*w*MelPop cells mapping to CFAV, despite being observed in the parental Aag2 cells (Fig. 1a). The majority of CFAV small RNAs in the parental Aag2 cells were 21 nt in size, with similar amounts mapping to the genome and the antigenome. In contrast, small RNAs mapping against PCLV were identified in Aag2 cells and Aag2*w*MelPop cells, with a higher percentage in the Aag2*w*MelPop cells (Fig. 1b). The majority of PCLV small RNAs were 26–30 nt, mapped to the anti-genome and had sequence specificities seen for ping-pong-derived PIWI-interacting RNAs (piRNAs) (adenine at positionp 10, A_10_, and uridine at position 1, U_1_) (Fig. S1, available in the online Supplementary Material). The S-segment could be considered as the highest producer of PCLV-specific small RNAs, followed by the L- and M-segments. For the S- and M-segments, a bias could be observed for small RNAs of 26–30 nt mapping mainly to the anti-genome. For the L-segment, similar amounts of small RNAs mapping to the genome/anti-genome were detected with a slight bias for the genome (Fig. 1c). Small RNAs of 26–30 nt mapping to the genome and antigenome of CFAV were detected only in parental Aag2 cells and were absent from Aag2*w*MelPop cells (Fig. 1a). These 26–30 nt RNAs contained the U1 bias, but lacked the A10 bias (Fig. S2). The small number of CFAV-specific small RNAs 26–30 nt in length meant it was not possible to analyse the sequence logos for the CFAV-specific sequences in Aag2*w*MelPop cells. The presence or absence of *w*MelPop, as well as PCLV and CFAV in these cells, was determined by reverse transcription PCR (RT-PCR) (Fig. 1d). These data suggested that wMelPop reduces or even clears CFAV infection in persistently infected Aag2 cells, but has no or little effect on PCLV.

### Effect of *Wolbachia* on persistent or acute ISV infection in Aag2 cells

**Fig. 2.**
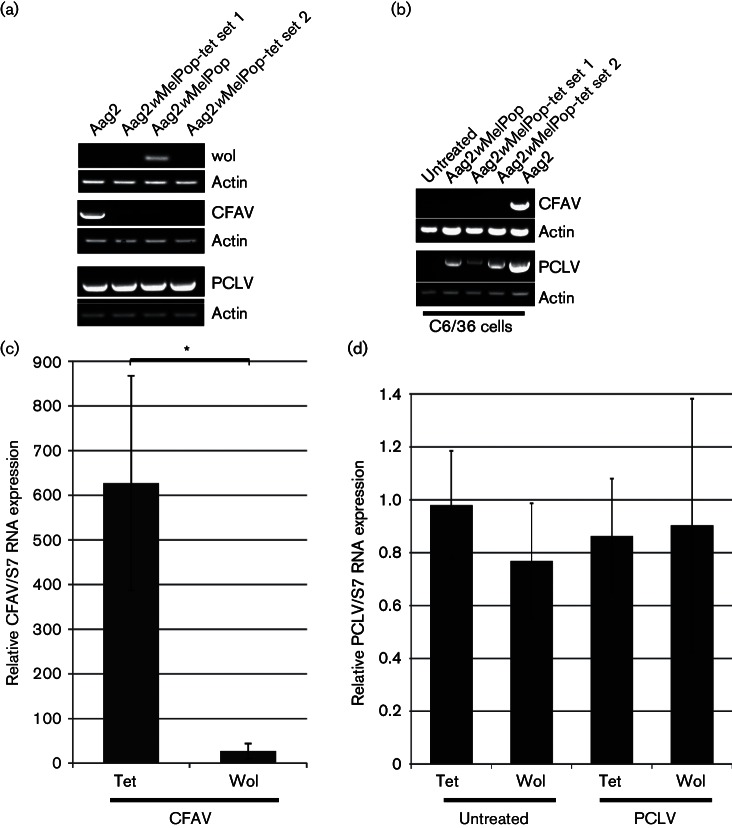
Effect of *w*MelPop on CFAV and PCLV infection in Aag2 cells. (a) Detection of CFAV, *Wolbachia* or PCLV in Aag2, Aag2*w*MelPop and two different cultures of Aag2*w*MelPop cells treated with tetracycline (Aag2*w*MelPop-tet sets 1 and 2) cells by RT-PCR. Actin was used as loading control. (b) Detection of CFAV or PCLV in C6/36 cells incubated with supernatant of Aag2, Aag2*w*MelPop or Aag2*w*MelPop treated with tetracycline (two different cultures, Aag2*w*MelPop-tet sets 1 and 2) by RT-PCR. Actin was used as a loading control. (c) Quantification of CFAV RNA in Aag2*w*MelPop (Wol) or Aag2*w*MelPop treated with tetracycline (Tet) cells after incubation with Aag2 supernatant containing CFAV by SYBR Green. S7 was used as internal control. Relative RNA expression is represented as (CFAV/S7). Error bars show sem from three independent experiments. (d) Quantification of PCLV RNA in Aag2*w*MelPop (Wol) or Aag2*w*MelPop treated with tetracycline (Tet) cells, either after incubation with Aag2 supernatant harbouring PCLV or untreated by SYBR Green. S7 was used as an internal control. Relative RNA expression is represented as (PCLV/S7) and mock-infected tetracycline cells were set to 1. Error bars show sem from three independent experiments. *P≤0.05.

The presence of active PCLV production/infection in Aag2 and Aag2*w*MelPop cells was further confirmed by RT-PCR and was also detected following the transfer of supernatant from these cells to C6/36 cells, resulting in PCLV-positive C6/36 cells (Fig. 2a, b). CFAV was easily detected by RT-PCR in Aag2 cells, as well as in C6/36 cells incubated with Aag2 supernatant, in contrast to Aag2*w*MelPop or C6/36 cells incubated with Aag2*w*MelPop supernatant (Fig. 2a, b). To determine if the presence of *w*MelPop in Aag2 cells cured the cells from CFAV infection or just strongly inhibited CFAV replication/infection, Aag2*w*MelPop cells were treated with tetracycline over several passages, resulting in the loss of *Wolbachia*. The absence of *Wolbachia* in Aag2*w*MelPop-tetracyline-treated cells (called Aag2*w*MelPop-tet) was confirmed by RT-PCR (Fig. 2a). Similar to what is seen in the parental Aag2*w*MelPop cells, no CFAV could be detected in Aag2*w*MelPop-tet cells (Fig. 2a), even when a different region of the CFAV genome was used for detection (Fig. S3a) or in C6/36 cells incubated with Aag2*w*MelPop-tet supernatant (Fig. 2b). In contrast, PCLV was detected in each of these samples (Fig. 2a, b). This suggested that *w*MelPop transinfection cures Aag2 cells of persistent CFAV infection, but has no effect on PCLV. To exclude the possibility that tetracycline treatment *per se* inhibits CFAV, Aag2 cells were treated with tetracycline and CFAV levels were monitored over time. No effect on CFAV could be detected in tetracycline-treated Aag2 cells compared to untreated cells (Fig. S3b).

To determine whether *w*MelPop has a similar effect on acute ISV infection, Aag2*w*MelPop and Aag2*w*MelPop-tet cells were incubated with Aag2 supernatant containing both CFAV and PCLV, and viral RNA detected by quantitative RT-PCR (Fig. 2c, d, respectively). Significantly less CFAV RNA was detected in Aag2*w*MelPop compared to Aag2*w*MelPop-tet cells. In contrast, no significant difference in PCLV RNA was observed under any of the test conditions.

In summary, these results show that *w*MelPop can inhibit CFAV infection in Aag2 cells, regardless of whether it is an acute or persistent infection, even resulting in total loss of CFAV in case of persistently infected cells. In contrast, no effect of PCLV was observed by *w*MelPop in Aag2 cells.

## Discussion

*Wolbachia* endosymbionts have been studied for their ability to restrict RNA virus infection in *Drosophila* and *A. aegypti* mosquitoes, as well as their derived cell lines (Kean *et al.*, 2015; Rainey *et al.*, 2014). Little is known about the effects mediated by *Wolbachia* to induce antiviral activity, although density has been reported to be important (Osborne *et al.*, 2009, 2012). Moreover, *Wolbachia* has recently been shown to inhibit early events during viral infection (Rainey *et al.*, 2016). Over the last decade, a variety of ISVs have been discovered in mosquitoes and for some of them an interaction with mosquito-borne viruses has been reported that may be either beneficial or disadvantageous for these viruses (Bolling *et al.*, 2015; Kean *et al.*, 2015). However, nothing is known about the effect of *Wolbachia* transinfection on ISVs present in mosquitoes and whether there is a difference in the interaction depending on the virus (e.g. positive- versus negative-strand RNA virus). Transinfection of *w*MelPop into *A. aegypti*-derived Aag2 cells resulted in the inhibition and even clearance of persistent CFAV infection in these cells, broadening the antiviral activity of *Wolbachia* from acute infection to persistent infection. This could also be observed at the level of small RNA production, which was produced in Aag2 cells but not *w*MelPop Aag2 cells. Similar antiviral effects by *Wolbachia* were observed when these cured cells were freshly infected with acute CFAV infection. In contrast, no effect on PCLV persistent infection in these cells was observed after *w*MelPop transinfection; in addition, superinfection of PCLV in previously transinfected *w*MelPop cells resulted in no difference in PCLV replication. As expected from these results, small RNAs against PCLV were produced in both Aag2 and *w*MelPop Aag2 cells.

CFAV-specific small RNAs showed a bias for 21 nt, the typical size of Dicer-2-produced small interfering RNAs, as previously reported for CFAV (Scott *et al.*, 2010) and other arthropod-borne flaviviruses (West Nile virus and DENV). In contrast, PCLV-specific small RNAs were mainly 26–30 nt in size, had a bias for the anti-genome and showed sequence-specific features for ping-pong-derived piRNAs (A_10_ and U_1_ bias) (Fig. S1). Similar results have been reported for other arthropod-borne bunyaviruses (Léger *et al.*, 2013; Schnettler *et al.*, 2013b), specifically for Rift Valley fever virus infection at later time points of infection (Léger *et al.*, 2013). Interestingly, CFAV small RNAs of length 26–30 nt show the classic ping-pong signature of U_1_ bias in the positive (genome) orientation, but lack the A_10_ bias in the negative (anti-genome) orientation (Fig. S2). This raises the question whether these small RNAs are in fact piRNAs, whether just one type of piRNAs is produced in CFAV infection of Aag2 cells or whether some small RNAs are products of some other RNA decay pathway.

These results illustrated a difference in the ability of the endosymbiont to interfere with persistently infecting ISVs from different families. Until now *Wolbachia* has only been reported to have an antiviral effect against positive-strand RNA viruses during an acute infection (Frentiu *et al.*, 2014; Martinez *et al.*, 2014; Rainey *et al.*, 2014, 2016), and the lack of effect of *Wolbachia* on PCLV is the first study to look at the interaction with a negative-strand RNA virus. Whether the observed lack of antiviral activity by *Wolbachia* is PCLV specific, or could be broadened to other negative-strand RNA viruses, still requires investigation. No antiviral effect was observed when persistently PCLV-infected and *w*MelPop-positive cells were superinfected with PCLV. It is not yet known whether this is due to the inability of *w*MelPop to inhibit PCLV infection, even at an acute stage of infection, or due to the inability of Aag2 cells to be superinfected with PCLV. Nonetheless, this raises some important questions for the field. For example, is *Wolbachia-*mediated inhibition limited to certain virus families and, if so, why is this the case? Could this be linked with the different small RNA profiles observed for flaviviruses versus bunyaviruses? How does this drive evolution of arboviruses or ISVs following the artificial introduction of *Wolbachia* into vector mosquitoes? What are the interactions between *Wolbachia* and ISVs and how do they influence vector competence in ISV-infected mosquitoes? Moreover, could it, for example during larger outbreaks involving many arboviruses, channel certain types of mosquito-borne pathogens and result in preferential amplification? Co-infection studies in mosquito systems with different families of arboviruses as well as ISVs are required to answer such questions.

In summary, *w*MelPop was able to efficiently inhibit persistent and acute infection of the positive-strand RNA insect-specific CFAV in Aag2 cells, but had no effect on persistent infection by the negative-strand RNA PCLV. Future research will have to investigate the effect of *Wolbachia* transinfection on other ISVs, as well as its effect on the complex interplay among ISVs, arboviruses and the mosquito vector, and how this influences/changes vector competence to different mosquito-borne viruses.

## Methods

### Cells and viruses.

*A. aegypti*-derived Aag2 wt, *w*MelPop-transinfected or *w*MelPop-transinfected and treated with tetracycline were maintained in Mitsuhashi and Maramorosch/Schneider’s (50 : 50) media supplemented with 10 % FCS and 10 % tryptose phosphate broth and PenStrep at 26 °C. Aag2- and *w*MelPop-transinfected cells were received from S. O'Neill and have been described previously (Mayoral *et al.*, 2014). Aag2*w*MelPop-tet cells were produced by passaging Aag2*w*MelPop cells with 10 µg ml^−1^ tetracycline for four passages and maintained as described. C6/36 cells were maintained in L15 medium supplemented with 10 % FCS and 10 % tryptose phosphate broth and PenStrep at 28 °C. CFAV and PCLV were derived from Aag2 wt supernatant.

### Reverse transcription, PCR and quantitative RT-PCR.

RT-PCR was performed with total RNA (1500 ng) isolated using TRIzol (Invitrogen), Superscript III and oligo-dT primer, according to the manufacturer’s protocol. CFAV, PCLV, *Wolbachia* and actin were detected and amplified by PCR (2 µl of the cDNA reaction) using corresponding primers [PCLV-N-FW: CAGTTAAAGCATTTAATCGTATGATAA, PCLV-N-RV: CACTAAGTGTTACAGCCCTTGGT, CFAV (3359 nt)-FW: GTTGACGACATATTGAAGAGATACG, CFAV (4060)-RV: GCCAAGGATACAGTCCAAAAC, CFAV-3UTR-FW: TAGACGTGATCGAATAGAGCCG, CFAV-3UTR-RV: GCGCATCTATGGTATAGAAAAGATAAT or as described previously (Rainey *et al.*, 2016; Schnettler *et al.*, 2013a)]. Quantitative detection of CFAV, PCLV and the housekeeping gene *S7* was performed using specific primers [PCLV-N-qRT-FW: ATAGTGTGGGACGAGGAGGG, PCLV-N-qRT-RV: AGGTGCCAACAGGAAACACT, CFAV-qRT-FW: CTGATGTGCGTGCAGTTCTT, CFAV-qRT-RV: CACAACGGTAGCGAGAGACA or as described previously (McFarlane *et al.*, 2014)], SYBR Green Master Mix (Applied Biosystems) and an ABI7500 Fast cycler according to manufacturer’s protocol.

### Virus infection.

Aag2*w*MelPop or Aag2*w*MelPop-tet cells were incubated with 200 µl Aag2 supernatant for 24 h, followed by 3× PBS washes and addition of fresh culture medium. RNA was isolated at 48 h post-infection.

### Small RNA analysis.

Small RNA reads from Aag2 (SRR1174240 and SRR1174241) and Aag2*w*MelPop cells (SRR1174242 and SRR1174243) published previously (Mayoral *et al.*, 2014) were re-analysed. The datasets were downloaded from the SRA database and FastqQ reads were extracted using SRA toolkit. Using blastn, these reads were mapped to the CFAV (NCBI accession number NC_001564.1) and PCLV (NCBI accession numbers KR003786.1, KR003784.1 and KR003785.1 correspond to L, M and S segments, respectively) genome and anti-genome. Hits that matched and 20–30 nt with one maximum mismatch were taken for later analysis. These hits were further categorized into two groups, mapping to both the genome and the anti-genome.

## Supplementary Data

2615 Supplementary File 1
