# Supplementary material for: Wolbachia restricts insect-specific flavivirus infection in Aedes aegypti cells
Source: J Gen Virol. 2016 Nov 10;97(11):3024–9. doi: 10.1099/jgv.0.000617 (PMC5120408; doi:10.1099/jgv.0.000617)
Supplement: Supplementary File 1 [file jgv-97-3024-s001.pdf]

Journal of General Virology

Wolbachia restricts insect specific flavivirus infection in Aedes aegypti cells.

--Manuscript Draft--

**Manuscript Number:**	JGV-D-16-00322R1
**Full Title:**	Wolbachia restricts insect specific flavivirus infection in Aedes aegypti cells.
**Short Title:**	Wolbachia restricts insect specific viruses
**Article Type:**	Standard
**Section/Category:**	Insect Viruses - RNA
**Corresponding Author:**	Esther Schnettler<br>Bernhard Nocht Institute for Tropical Medicine<br>Hamburg, GERMANY
**First Author:**	Esther Schnettler
**Order of Authors:**	Esther Schnettler
	Vatipally B. Sreenu
	Timothy Mottram
	Melanie McFarlane
**Abstract:**	Mosquito-borne viruses are known to cause disease in humans and livestock and are often difficult to control due the lack of specific antivirals and vaccines. The Wolbachia endosymbiont has been widely studied for its ability to restrict positive strand RNA virus infection in mosquitoes, although little is known about the precise antiviral mechanism. In recent years, a variety of insect-specific viruses have been discovered in mosquitoes and an interaction with mosquito-borne viruses have been reported for some of them; however nothing is known about the effect of Wolbachia on insect specific virus infection in mosquitoes. Here, we show that transinfection of the drosophila derived wMelPop Wolbachia strain into Ae. aegypti-derived cells resulted in inhibition and even clearance of the persistent cell fusing agent flavivirus infection in these cells. This, broadens the antiviral activity of Wolbachia from acute infections to persistent infections and from arboviruses to mosquito-specific viruses. In contrast, no effect on the Phasi-Charoen-like bunyavirus persistent infection in these cells was observed, suggesting a difference in Wolbachia inhibition between positive and negative strand RNA viruses.

Powered by Editorial Manager® and ProduXion Manager® from Aries Systems Corporation

Manuscript Including References (Word document) Click here to download Manuscript Including References (Word document) Schnettler-Vatipally-Wolbachia-04092016-

***Wolbachia* restricts insect specific flavivirus infection in *Aedes aegypti* cells.**

Esther Schnettler[1,2#], Vatipally B. Sreenu[1], Timothy Mottram[1] and Melanie McFarlane[1]

[1]MRC-University of Glasgow Centre for Virus Research, Glasgow G61 1QH, Scotland, UK

[2]Current address: BNITM, Bernhard-Nocht-Strasse 74, 20359 Hamburg, Germany

[#]Corresponding author

Email: Schnettler@bnitm.de,

Phone: +49 40 42818-0

Running title: *Wolbachia* restricts insect specific viruses

Key words: *Wolbachia*, insect specific virus, CFAV, PCLV, *Ae. aegypti*

Abstract

Mosquito-borne viruses are known to cause disease in humans and livestock and are often difficult to control due the lack of specific antivirals and vaccines. The *Wolbachia* endosymbiont has been widely studied for its ability to restrict positive strand RNA virus infection in mosquitoes, although little is known about the precise antiviral mechanism. In recent years, a variety of insect-specific viruses have been discovered in mosquitoes and an interaction with mosquito-borne viruses have been reported for some of them; however, nothing is known about the effect of *Wolbachia* on insect specific virus infection in mosquitoes. Here we show that transinfection of the drosophila derived *w*MelPop *Wolbachia* strain into *Ae. aegypti*-derived cells resulted in inhibition and even clearance of the persistent cell fusing agent flavivirus infection in these cells. This broadens the antiviral activity of *Wolbachia* from acute infections to persistent infections and from arboviruses to mosquito-specific viruses. In contrast, no effect on the Phasi-Charoen-like bunyavirus persistent infection in these cells was observed, suggesting a difference in *Wolbachia* inhibition between positive and negative strand RNA viruses.

Introduction

Arboviruses are comprised of human and animal pathogens that are transmitted via blood feeding arthropod vectors, including mosquitoes. Due to the lack of efficient antivirals and vaccines against most of these viruses, vector control is an important intervention strategy to reduce the impact of these viruses on human and animal health (Kean et al., 2015, Weaver and Reisen, 2010). In recent years the use of the endosymbiotic intracellular bacterium, *Wolbachia* has been a well studied approach to control arbovirus transmission by mosquitoes and in particular by *Aedes aegypti* (Rainey et al., 2014, Iturbe-Ormaetxe et al., 2011). *Wolbachia* was first shown to confer resistance to RNA viruses in *Drosophila*-virus systems (Hedges et al., 2008, Teixeira et al., 2008). Later, transinfection of drosophila derived *Wolbachia* into *Ae. aegypti* (which is not known to naturally harbour these endosymbionts) or its derived cell lines resulted in resistance to the important mosquito-borne dengue (DENV) and chikungunya viruses (Walker et al., 2011, Moreira et al., 2009). This has resulted in successful field trials of *Ae. aegypti* transinfected with *Wolbachia*, proving its ability to reduce DENV transmission in natural settings (Frentiu et al., 2014). Moreover, *Wolbachia* can be stably maintained in nature, as crosses between non-infected females and infected males do not result in any offspring (Hoffmann et al., 2011, Hoffmann et al., 2014). This unique feature is called cytoplasmic incapability (McMeniman et al., 2009) and gives a reproductive advantage to infected female mosquitoes, resulting in the spread of *Wolbachia* through the mosquito population (Sinkins, 2004).

The mechanism(s) of virus inhibition through *Wolbachia* is not known. Inhibition has been linked to *Wolbachia* density, with the resistant phenotype observed only with *Wolbachia* strains producing high concentrations of bacteria in infected cells (Osborne et al., 2012, Osborne et al., 2009). Recent findings, show the ability of *Wolbachia* to interfere with early events in virus replication, suggesting an intrinsic mechanism for viral resistance (Rainey et al., 2016).

It should be noted that *Wolbachia*-mediated virus resistance has only been reported for positive-stranded RNA viruses and no resistance has yet been reported for negative-stranded RNA viruses (Rainey et al., 2014), which include a variety of important mosquito-borne viruses such as Rift Valley fever virus (*Bunyaviridae*).

Further to arboviruses, mosquitoes have also been shown to be infected with additional viruses, called insect specific viruses (ISVs) as they replicate exclusively in insect cells. The list

of ISVs is steadily increasing through novel identification methods, including next generation sequencing. ISVs belong to different virus families/genera, including the *Bunyaviridae* and *Flaviviridae* families, which also include important arboviruses. ISVs belonging to the Flavivirus genus share sequence similarities with their arbovirus counterparts, but cluster as a single defined group suggesting independent evolution. In contrast, ISVs belonging to the *Bunyaviridae*, cluster into several defined groups across the virus family (Bolling et al., 2015, Marklewitz et al., 2015). ISV infections, at least in cell culture, normally result in initial cytopathic effect (CPE), followed by progression into a persistent, non-cytopathic infection (Bolling et al., 2015, Marklewitz et al., 2015).

The increasing numbers of ISVs identified in mosquitoes and derived cells suggest that a large number of mosquitoes in the wild are naturally infected with ISVs and that vertical transmission is the main infection and maintenance route. Thereby one can expect that mosquitoes in the wild can be infected by several viruses, including ISVs and/or arboviruses. Moreover, the interaction between ISV and arbovirus infections (either co-infected or sequentially infected) results in either inhibition or increased replication/infection of one of the viruses (Kean et al., 2015). It is suggested that such interactions could partly define vector competence of a mosquito in the wild to a given arbovirus.

No information is available at the moment about the interaction of *Wolbachia* with these ISVs or what effect *Wolbachia* transinfection could have on mosquitoes already persistently infected with RNA viruses. The inhibitory effect of *Wolbachia* on RNA viruses has only been investigated in light of an acute virus infection following a persistent *Wolbachia* transinfection (Rainey et al., 2014).

In order to address these questions and to understand if *Wolbachia* interacts with acute or persistent infections of ISVs we have used the *Ae. aegypti*-derived Aag2 cell line previously transinfected with the drosophila derived *Wolbachia* strain *w*MelPop (known to grow to high titres and mediate DENV resistance) (Hedges et al., 2008, Teixeira et al., 2008) to investigate the effect of *Wolbachia* on two ISVs, known to be present in Aag2 cells and belonging to different families: positive strand RNA cell fusing agent virus (CFAV, *Flaviviridae*) (Scott et al., 2010) and the negative strand RNA Phasi-charoen-like bunyavirus (PCLV, *Bunyaviridae*)

(Maringer et al., 2015). Our results show that *Wolbachia* can refer resistance to CFAV infection independently of the time of *Wolbachia* transinfection. In contrast, no viral inhibition by *Wolbachia* was observed for PCLV in these experiments.

Results

Effect of *Wolbachia* on small RNA production in Aag2 cells

Aag2 cells can be stably transinduced with the *w*MelP strain of Drosophila, resulting in a reduction of small RNAs in the cytoplasm due to inhibition of small RNA transport from the nucleus to the cytoplasm (Mayoral et al. 2014). Aag2 cells are known to be persistently infected with the insect-specific flavivirus, CFAV and as result produce CFAV-specific small RNAs (Scott et al., 2010). Recently it has also been reported that Aag2 cells produce transcripts and proteins from another ISV, PCLV (suggesting a persistent infection) (Maringer et al., 2015). However, it is not yet known if this is due to an active virus infection. This virus has also been recently discovered in wild mosquitoes in Brazil (Aguiar et al., 2015). Therefore we re-analysed the previously reported small RNA data of Aag2 and Aag2*w*MelPop cells (Mayoral et al., 2014) and mapped them to CFAV or PCLV. Nearly no small RNA reads were detected in Aag2*w*MelPop cells mapping to CFAV, despite being observed in the parental Aag2 cells (Fig. 1a). The majority of CFAV small RNAs in the parental Aag2 cells were 21 nts in size with similar amounts mapping to the genome and the antigenome. In contrast, small RNAs mapping against PCLV were identified in Aag2 cells and Aag2*w*MelPop cells with a higher percentage in the Aag2*w*MelPop cells (Fig. 1b). The majority of PCLV small RNAs were 26-30nts, mapped to the antigenome and had sequence specifisities seen for ping-pong derived piRNAs (adenine at position 10, A_{10}, and Uridine at position 1, U_1) (Fig. S1). The S segment could be considered as highest producer of PCLV specific small RNAs, followed by the L- and the M-segment. For the S- and M-segment a bias could be observed for small RNAs of 26-30 nts mapping mainly to the antigenome. For the L-segment similar amounts of small RNAs mapping to the genome/antigenome were detected with a slight bias for the genome (Fig. 1c). Small RNAs of 26-30nts mapping to the genome and antigenome of CFAV were detected only in parental Aag2 cells and were absent from Aag2*w*MelPop cells (Fig. 1a). These 26-30nt RNAs contained the U_1 bias but lacked the A_{10} bias (Fig. S2). The small number of CFAV specific small RNAs of 26-30nt in length meant it was not possible to

analyse the sequence logos for the CFAV specific sequences in Aag2*w*Melpop cells.The presence or absence of *w*MelPop as well as PCLV and CFAV in these cells was determined by RT-PCR (Fig. 1d). This data suggested that *w*MelPop reduces or even clears CFAV infection in persistently infected Aag2 cells, but has no or little effect on PCLV.

Effect of *Wolbachia* on persistent or acute ISV infection in Aag2 cells

The presence of active PCLV production/infection in Aag2 and Aag2*w*MelPop cells was further confirmed by RT-PCR and was also detected following the transfer of supernatant from these cells to C6/36 cells, resulting in PCLV positive C6/36 cells (Fig. 2a and b). CFAV could be easily detected by RT-PCR in Aag2 cells as well as in C6/36 cells incubated with Aag2 supernatant in contrast to Aag2*w*MelPop or C6/36 cells incubated with Aag2*w*MelPop supernatant (Fig. 2a and b). To determine if the presence of *w*MelPop in Aag2 cells cured the cells from CFAV infection or just strongly inhibited CFAV replication/infection, Aag2*w*MelPop cells were treated with tetracycline over several passages resulting in the loss of *Wolbachia*. The absence of *Wolbachia* in Aag2*w*MelPop-tetracyline treated cells (called Aag2*w*MelPop-tet) was confirmed by RT-PCR (Fig. 2a). Similar to what is seen in the parental Aag2wMelPop cells no CFAV could be detected in Aag2*w*MelPop-tet cells (Fig. 2a), even if a different region of the CFAV genome was used for detection (Fig. S3a), or in C6/36 cells incubated with Aag2*w*MelPop-tet supernatant (Fig. 2b). In contrast, PCLV was detected in each of these samples (Fig. 2a and b). This suggested that *w*MelPop transinfection cures Aag2 cells from the persistent CFAV infection, but has no effect on PCLV. To exclude that tetracycline treatment by itself inhibits CFAV; Aag2 cells were treated with tetracycline and CFAV levels were monitored over time. No effect on CFAV could be detected in tetracycline treated Aag2 cells compared to untreated cells (S3b).

To determine if *w*MelPop has a similar effect on an acute ISV infection, Aag2*w*MelPop and Aag2*w*MelPop-tet cells were incubated with Aag2 supernatant containing both CFAV and PCLV; and viral RNA detected by qRT-PCR (Fig. 2c and 2d, respectively). Significantly less CFAV RNA was detected in Aag2*w*MelPop compared to Aag2*w*MelPop-tet cells. In contrast, no significant difference in PCLV RNA was observed under any of the used conditions.

In summary, these results show that *w*MelPop can inhibit CFAV infection in Aag2 cells, regardless of whether it is an acute or persistent infection, even resulting in total loss of CFAV in case of persistently infected cells. In contrast, no effect of PCLV was observed by

*w*MelPop in Aag2 cells.

Discussion

Wolbachia endosymbionts have been studied for their ability to restrict RNA virus infection in drosophila and *Ae. aegypti* mosquitoes as well as their derived cell lines (Kean et al., 2015, Rainey et al., 2014). Little is known about the effects mediated by *Wolbachia* to induce antiviral activity, although density has been reported to be important (Osborne et al., 2012, Osborne et al., 2009). Moreover, *Wolbachia* has recently been shown to inhibit early events during viral infection (Rainey et al., 2016). Over the last decade a variety of ISVs have been discovered in mosquitoes and for some of them an interaction with mosquito-borne viruses have been reported which may be either beneficial or disadvantageous for these viruses (Bolling et al., 2015, Kean et al., 2015). However, nothing is known about the effect of *Wolbachia* transinfection on ISVs present in mosquitoes and if there is a difference in the interaction depending on the virus (e.g. positive versus negative strand RNA virus). Transinfection of *w*MelPop into *Ae. aegypti*-derived Aag2 cells resulted in the inhibition and even clearance of the persistent CFAV infection in these cells, broadening the antiviral activity of *Wolbachia* from acute infections to persistent infections. This could also be observed on the level of small RNA production, which were produced in Aag2 cells but not *w*MelPop Aag2 cells. Similar antiviral effects by *Wolbachia* were observed if these cured cells were freshly infected with an acute CFAV infection. In contrast, no effect on PCLV persistent infection in these cells was observed after *w*MelPop transinfection; in addition, superinfection of PCLV in the already transinfected *w*MelPop cells resulted in no difference in PCLV replication. As expected from these results, small RNAs against PCLV were produced in both Aag2 and *w*MelPop Aag2 cells.

CFAV-specific small RNAs showed a bias for 21 nts, the typical size of Dicer-2 produced siRNAs, as previously reported for CFAV (Scott et al., 2010) and other arthropod-borne flaviviruses (WNV, DENV). In contrast, PCLV specific small RNAs were mainly in the size of 26-30 nts, had a bias for the antigenome and showed sequence specific features for ping-pong derived piRNAs (A_{10} and U_1 bias) (Fig. S1). Similar results have been reported for other arthropod-borne bunyaviruses (Schnettler et al., 2013b, Leger et al., 2013); specifically for Rift valley fever virus infection at later time points of infection (Leger et al., 2013). Interestingly, CFAV small RNAs of 26-30nt in length show the classic ping-pong signature of

U_1 bias in the positive (genome) orientation but lack the A_{10} bias in the negative (antigenome) orientation (Fig S2). This raises the question of whether these small RNAs are in fact piRNAs, or whether just one type of piRNAs are produced in CFAV infection of Aag2 cells, or if some small RNAs are products of some other RNA decay pathway.These results illustrated a difference in the ability of the endosymbiont to interfere with persistently infecting ISVs from different families. Until now *Wolbachia* has only been reported to have an antiviral effect against positive strand RNA viruses during an acute infection (Frentiu et al., 2014, Martinez et al., 2014, Rainey et al., 2016, Rainey et al., 2014) and the lack of effect of *Wolbachia* on PCLV is the first study to look at the interaction with a negative strand RNA virus. Whether the observed lack of antiviral activity by *Wolbachia* is PCLV specific or could be broadened to other negative strand RNA viruses still requires investigation. No antiviral effect was observed if persistently PCLV infected and *w*MelPop positive cells were superinfected with PCLV. It is not yet known if this is due to the inability of *w*MelPop to inhibit PCLV infection, even at an acute stage of infection, or due to the inability of Aag2 cells to be superinfected with PCLV. Nonetheless, this raises some important questions for the field. For example, is *Wolbachia* mediated inhibition limited to certain virus families and if yes why is this the case? Could this be linked with the different small RNA profiles observed for flaviviruses versus bunyaviruses? How does this drive evolution of arboviruses or ISVs following the artificial introduction of *Wolbachia* into vector mosquitoes? What are the interactions between *Wolbachia* and ISVs and how do they influence vector competence in ISV infected mosquitoes? Moreover, could it, for example during larger outbreaks involving many arboviruses, channel certain types of mosquito-borne pathogens and result in preferential amplification? Co-infection studies in mosquito systems with different families of arboviruses as well as ISVs are required to answer such questions.

In summary, *w*MelPop is able to efficiently inhibit persistent and acute infection of the positive strand RNA insect-specific CFAV in Aag2 cells but has no effect on the persistent infection by the negative strand RNA PCLV. Future research will have to investigate what effect *Wolbachia* transinfection has on other ISVs as well as its effect on the complex interplay between ISVs, arboviruses and the mosquito vector and how this influences/changes vector competence to different mosquito-borne viruses.

Methods

Cells and viruses

Ae. aegypti-derived Aag2 wt, *w*MelPop transinfected or *w*MelPop transinfected and treated with tetracycline were maintained in Mitsuhashi and Maramorosch/ Schneider's (50:50) media supplemented with 10% foetal calf serum and 10% tryptose phosphate broth and PenStrep at 26° C. Aag2 and *w*MelPop transinfected cells were received from S. O`Neill and have been previously described (Mayoral et al., 2014). Aag2*w*MelPop-tet cells were produced by passaging Aag2*w*MelPop cells with 10ug/ml tetracycline for 4 passages and maintained as described. C6/36 cells were maintained in L15 media supplemented with 10% foetal calf serum and 10% tryptose phosphate broth and PenStrep at 28° C. CFAV and PCLV were derived from Aag2 wt supernatant.

Reverse transcription, PCR and qRT-PCR

Reverse transcription (RT)-PCR was performed with total RNA (1500 ng) isolated using TRIzol (Invitrogen), Superscript III and oligo-dT primer, according to the manufacturer's protocol. CFAV, PCLV, *Wolbachia* and actin were detected and amplified by PCR (2 µl of the cDNA reaction) using corresponding primers (PCLV-N-FW: CAGTTAAAGCATTTAATCGTATGATAA; PCLV-N-RV: CACTAAGTGTTACAGCCCTTGGT; CFAV(3359 nt)-FW: GTTGACGACATATTGAAGAGATACG; CFAV(4060 nt)-RV: GCCAAGGATACAGTCCAAAAC; CFAV-3UTR-FW: TAGACGTGATCGAATAGAGCCG; CFAV-3UTR-RV: GCGCATCTATGGTATAGAAAAGATAAT or previously described (Rainey et al., 2016, Schnettler et al., 2013a)). Quantitative detection of CFAV, PCLV and the housekeeping gene S7 was performed using specific primers (PCLV-N-qRT-FW: ATAGTGTGGGACGAGGAGGG; PCLV-N-qRT-RV: AGGTGCCAACAGGAAACACT; CFAV-qRT-FW: CTGATGTGCGTGCAGTTCTT; CFAV-qRT-RV: CACAACGGTAGCGAGAGACA or as previously described (McFarlane et al., 2014)), SYBR green Mastermix (Abi) and an ABI7500 Fast cycler according to manufacturer's protocol.

Virus infection

Aag2*w*MelPop or Aag2*w*MelPop-tet cells were incubated with 200 µl Aag2 supernatant for 24 h; followed by 3x PBS washes and addition of fresh culture medium. RNA was isolated at 48 hours post infection.

Small RNA analysis

Small RNA reads from Aag2 (SRR1174240, and SRR1174241) and Aag2*w*MelPop cells (SRR1174242 and SRR1174243) published previously (Mayoral et al., 2014) were re-analysed. The datasets were downloaded from SRA database and FastqQ reads were extracted using SRA toolkit. Using blastn these reads were mapped to the CFAV (NCBI accession number NC_001564.1) and PCLV (NCBI accession numbers KR003786.1, KR003784.1 and KR003785.1 correspond to L, M and S segments respectively) genome and antigenome. Hits that were matching and 20nt to 30nt with one maximum mismatch were taken for later analysis. These hits were further categorised into two groups, mapping to the genome and mapping to anti-genome.

Acknowledgments

This work was supported by the MRC (MC_UU_12014). The authors want to thank Scott O`Neill for providing the Aag2 and Aag2*w*MelPop cells and members of the Kohl lab for critical reading of the manuscript.

References

AGUIAR, E. R., OLMO, R. P., PARO, S., FERREIRA, F. V., DE FARIA, I. J., TODJRO, Y. M., LOBO, F. P., KROON, E. G., MEIGNIN, C., GATHERER, D., IMLER, J. L. & MARQUES, J. T. 2015. Sequence-independent characterization of viruses based on the pattern of viral small RNAs produced by the host. *Nucleic Acids Res,* 43**,** 6191-206.

BOLLING, B. G., WEAVER, S. C., TESH, R. B. & VASILAKIS, N. 2015. Insect-Specific Virus Discovery: Significance for the Arbovirus Community. *Viruses,* 7**,** 4911-28.

FRENTIU, F. D., ZAKIR, T., WALKER, T., POPOVICI, J., PYKE, A. T., VAN DEN HURK, A., MCGRAW, E. A. & O'NEILL, S. L. 2014. Limited dengue virus replication in field-collected Aedes aegypti mosquitoes infected with Wolbachia. *PLoS Negl Trop Dis,* 8**,** e2688.

HEDGES, L. M., BROWNLIE, J. C., O'NEILL, S. L. & JOHNSON, K. N. 2008. Wolbachia and virus protection in insects. *Science,* 322**,** 702.

HOFFMANN, A. A., ITURBE-ORMAETXE, I., CALLAHAN, A. G., PHILLIPS, B. L., BILLINGTON, K., AXFORD, J. K., MONTGOMERY, B., TURLEY, A. P. & O'NEILL, S. L. 2014. Stability of the wMel Wolbachia Infection following invasion into Aedes aegypti populations. *PLoS Negl Trop Dis,* 8**,** e3115.

HOFFMANN, A. A., MONTGOMERY, B. L., POPOVICI, J., ITURBE-ORMAETXE, I., JOHNSON, P. H., MUZZI, F., GREENFIELD, M., DURKAN, M., LEONG, Y. S., DONG, Y., COOK, H., AXFORD, J., CALLAHAN, A. G., KENNY, N., OMODEI, C., MCGRAW, E. A., RYAN, P. A., RITCHIE, S. A., TURELLI, M. & O'NEILL, S. L. 2011. Successful establishment of Wolbachia in Aedes populations to suppress dengue transmission. *Nature,* 476**,** 454-7.

ITURBE-ORMAETXE, I., WALKER, T. & SL, O. N. 2011. Wolbachia and the biological control of mosquito-borne disease. *EMBO Rep,* 12**,** 508-18.

KEAN, J., RAINEY, S., MCFARLANE, M., DONALD, C., SCHNETTLER, E., KOHL, A. & PONDEVILLE, E. 2015. Fighting Arbovirus Transmission: Natural and Engineered Control of Vector Competence in Aedes Mosquitoes. *Insects,* 6**,** 236.

LEGER, P., LARA, E., JAGLA, B., SISMEIRO, O., MANSUROGLU, Z., COPPEE, J. Y., BONNEFOY, E. & BOULOY, M. 2013. Dicer-2- and Piwi-mediated RNA interference in Rift Valley fever virus-infected mosquito cells. *J Virol,* 87**,** 1631-48.

MARINGER, K., YOUSUF, A., HEESOM, K., FERNANDEZ-SESMA, A., MATTHEWS, D. A. & DAVIDSON, A. D. 2015. De novo 'proteomics informed by transcriptomics' analysis of Aedes aegypti cells identifies novel ORFs, active transposable elements and persistent viruses. *Conference paper*.

MARKLEWITZ, M., ZIRKEL, F., KURTH, A., DROSTEN, C. & JUNGLEN, S. 2015. Evolutionary and phenotypic analysis of live virus isolates suggests arthropod origin of a pathogenic RNA virus family. *Proc Natl Acad Sci U S A,* 112**,** 7536-41.

MARTINEZ, J., LONGDON, B., BAUER, S., CHAN, Y. S., MILLER, W. J., BOURTZIS, K., TEIXEIRA, L. & JIGGINS, F. M. 2014. Symbionts commonly provide broad spectrum resistance to viruses in insects: a comparative analysis of Wolbachia strains. *PLoS Pathog,* 10**,** e1004369.

MAYORAL, J. G., ETEBARI, K., HUSSAIN, M., KHROMYKH, A. A. & ASGARI, S. 2014. Wolbachia infection modifies the profile, shuttling and structure of microRNAs in a mosquito cell line. *PLoS One,* 9**,** e96107.

MCFARLANE, M., ARIAS-GOETA, C., MARTIN, E., O'HARA, Z., LULLA, A., MOUSSON, L., RAINEY, S. M., MISBAH, S., SCHNETTLER, E., DONALD, C. L., MERITS, A., KOHL, A. & FAILLOUX, A. B. 2014. Characterization of Aedes aegypti Innate-Immune Pathways that Limit Chikungunya Virus Replication. *PLoS Negl Trop Dis,* 8**,** e2994.

MCMENIMAN, C. J., LANE, R. V., CASS, B. N., FONG, A. W., SIDHU, M., WANG, Y. F. & O'NEILL, S. L. 2009. Stable introduction of a life-shortening Wolbachia infection into the mosquito Aedes aegypti. *Science,* 323**,** 141-4.

MOREIRA, L. A., ITURBE-ORMAETXE, I., JEFFERY, J. A., LU, G., PYKE, A. T., HEDGES, L. M., ROCHA, B. C., HALL-MENDELIN, S., DAY, A., RIEGLER, M., HUGO, L. E., JOHNSON, K. N., KAY, B. H., MCGRAW, E. A., VAN DEN HURK, A. F., RYAN, P. A. & O'NEILL, S. L. 2009. A Wolbachia symbiont in Aedes aegypti limits infection with dengue, Chikungunya, and Plasmodium. *Cell,* 139**,** 1268-78.

OSBORNE, S. E., ITURBE-ORMAETXE, I., BROWNLIE, J. C., O'NEILL, S. L. & JOHNSON, K. N. 2012. Antiviral protection and the importance of Wolbachia density and tissue tropism in Drosophila simulans. *Applied and environmental microbiology,* 78**,** 6922-9.

OSBORNE, S. E., LEONG, Y. S., O'NEILL, S. L. & JOHNSON, K. N. 2009. Variation in antiviral protection mediated by different Wolbachia strains in Drosophila simulans. *PLoS Pathog,* 5**,** e1000656.

RAINEY, S. M., MARTINEZ, J., MCFARLANE, M., JUNEJA, P., SARKIES, P., LULLA, A., SCHNETTLER, E., VARJAK, M., MERITS, A., MISKA, E. A., JIGGINS, F. M. & KOHL, A. 2016. Wolbachia Blocks Viral Genome Replication Early in Infection without a Transcriptional Response by the Endosymbiont or Host Small RNA Pathways. *PLoS Pathog,* 12**,** e1005536.

RAINEY, S. M., SHAH, P., KOHL, A. & DIETRICH, I. 2014. Understanding the Wolbachia-mediated inhibition of arboviruses in mosquitoes: progress and challenges. *J Gen Virol,* 95**,** 517-30.
SCHNETTLER, E., DONALD, C. L., HUMAN, S., WATSON, M., SIU, R. W., MCFARLANE, M., FAZAKERLEY, J. K., KOHL, A. & FRAGKOUDIS, R. 2013a. Knockdown of piRNA pathway proteins results in enhanced Semliki Forest virus production in mosquito cells. *J Gen Virol,* 94**,** 1680-9.
SCHNETTLER, E., RATINIER, M., WATSON, M., SHAW, A. E., MCFARLANE, M., VARELA, M., ELLIOTT, R. M., PALMARINI, M. & KOHL, A. 2013b. RNA interference targets arbovirus replication in Culicoides cells. *J Virol,* 87**,** 2441-54.
SCOTT, J. C., BRACKNEY, D. E., CAMPBELL, C. L., BONDU-HAWKINS, V., HJELLE, B., EBEL, G. D., OLSON, K. E. & BLAIR, C. D. 2010. Comparison of dengue virus type 2-specific small RNAs from RNA interference-competent and -incompetent mosquito cells. *PLoS Negl Trop Dis,* 4**,** e848.
SINKINS, S. P. 2004. Wolbachia and cytoplasmic incompatibility in mosquitoes. *Insect Biochemistry and Molecular Biology,* 34**,** 723-9.
TEIXEIRA, L., FERREIRA, A. & ASHBURNER, M. 2008. The bacterial symbiont Wolbachia induces resistance to RNA viral infections in Drosophila melanogaster. *PLoS Biol,* 6**,** e2.
WALKER, T., JOHNSON, P. H., MOREIRA, L. A., ITURBE-ORMAETXE, I., FRENTIU, F. D., MCMENIMAN, C. J., LEONG, Y. S., DONG, Y., AXFORD, J., KRIESNER, P., LLOYD, A. L., RITCHIE, S. A., O'NEILL, S. L. & HOFFMANN, A. A. 2011. The wMel Wolbachia strain blocks dengue and invades caged Aedes aegypti populations. *Nature,* 476**,** 450-3.
WEAVER, S. C. & REISEN, W. K. 2010. Present and future arboviral threats. *Antiviral Res,* 85**,** 328-45.

Fig. 1. Presence or absence of CFAV, PCLV and *w*MelPop in Aag2 and Aag2*w*MelPop cells. Size distribution of small RNA molecules mapping to the CFAV **(a)** or PCLV **(b)** genome (black)/ antigenome (grey) in *Ae. aegypti*-derived Aag2 or *w*MelPop transinfected Aag2 cells. **(c)** Size distribution of small RNA molecules mapping to the different segments of PCLV (S, M and L) genome/antigenome in *Ae. aegypti*-derived Aag2 or *w*MelPop transinfected Aag2 cells. **(d)** Detection of CFAV or PCLV in Aag2 and Aag2*w*MelPop cells by RT-PCR. Actin was used as loading control.

Fig. 2. Effect of *w*MelPop on CFAV and PCLV infection in Aag2 cells. (a) Detection of CFAV, *Wolbachia* or PCLV in Aag2, Aag2*w*MelPop and two different cultures of Aag2*w*MelPop cells treated with tetracycline (Aag2*w*MelPop-tet set 1 and 2) cells by RT-PCR. Actin was used as loading control. **(b)** Detection of CFAV or PCLV in C6/36 cells incubated with supernatant of Aag2, Aag2*w*MelPop or Aag2*w*MelPop treated with tetracycline (two different cultures, Aag2*w*MelPop-tet set 1 and 2) by RT-PCR. Actin was used as a loading control. **(c)** Quantification of CFAV RNA in Aag2*w*MelPop (Wol) or Aag2*w*MelPop treated with tetracycline (tet) cells after incubation with Aag2 supernatant containing CFAV by SYBR green. S7 was used as internal control. Relative RNA expression is represented as (CFAV/S7). Error bars show standard error of mean from three independent experiments. **(d)** Quantification of PCLV RNA in Aag2*w*MelPop (Wol) or Aag2*w*MelPop treated with tetracycline (tet) cells, either after incubation with Aag2 supernatant harbouring PCLV or untreated by SYBR green. S7 was used as an internal control. Relative RNA expression is represented as (PCLV/S7) and mock-infected tet cells were set to 1. Error bars show standard error of mean from three independent experiments. * is $p \leq 0.05$.

Supplementary data

Fig. S1: PCLV specific piRNAs in Aag2 cells.
Relative nucleotide frequency and conservation per position of 28 nt long small RNAs mapping to the genome and antigenome of PCLV in Aag2 and Aag2*w*MelPop cells are indicated. Sequence is represented as DNA.

Fig. S2: CFAV specific piRNAs in Aag2 cells.
Relative nucleotide frequency and conservation per position of 28 nt small RNAs mapping to the genome and antigenome of CFAV in Aag2 cells are indicated. Sequence is represented as DNA.

Fig. S3: Effect of tetracycline treatment on CFAV infection in Aag2 cells. (a) Detection of CFAV (two different primer pairs: 3`UTR or non-structural region) and *Wolbachia* in Aag2, Aag2*w*MelPop and Aag2*w*MelPop cells treated with tetracycline by RT-PCR. Actin was used as loading control. **(b)** Detection of CFAV (two different primer pairs; 3`UTR or non-structural region) in Aag2 or Aag2 cells treated with tetracycline cells at different time points (passage 1 top or passage 6 bottom) by RT-PCR. Actin was used as loading control.

Figure 1

Click here to download Figure Figure 1.tif

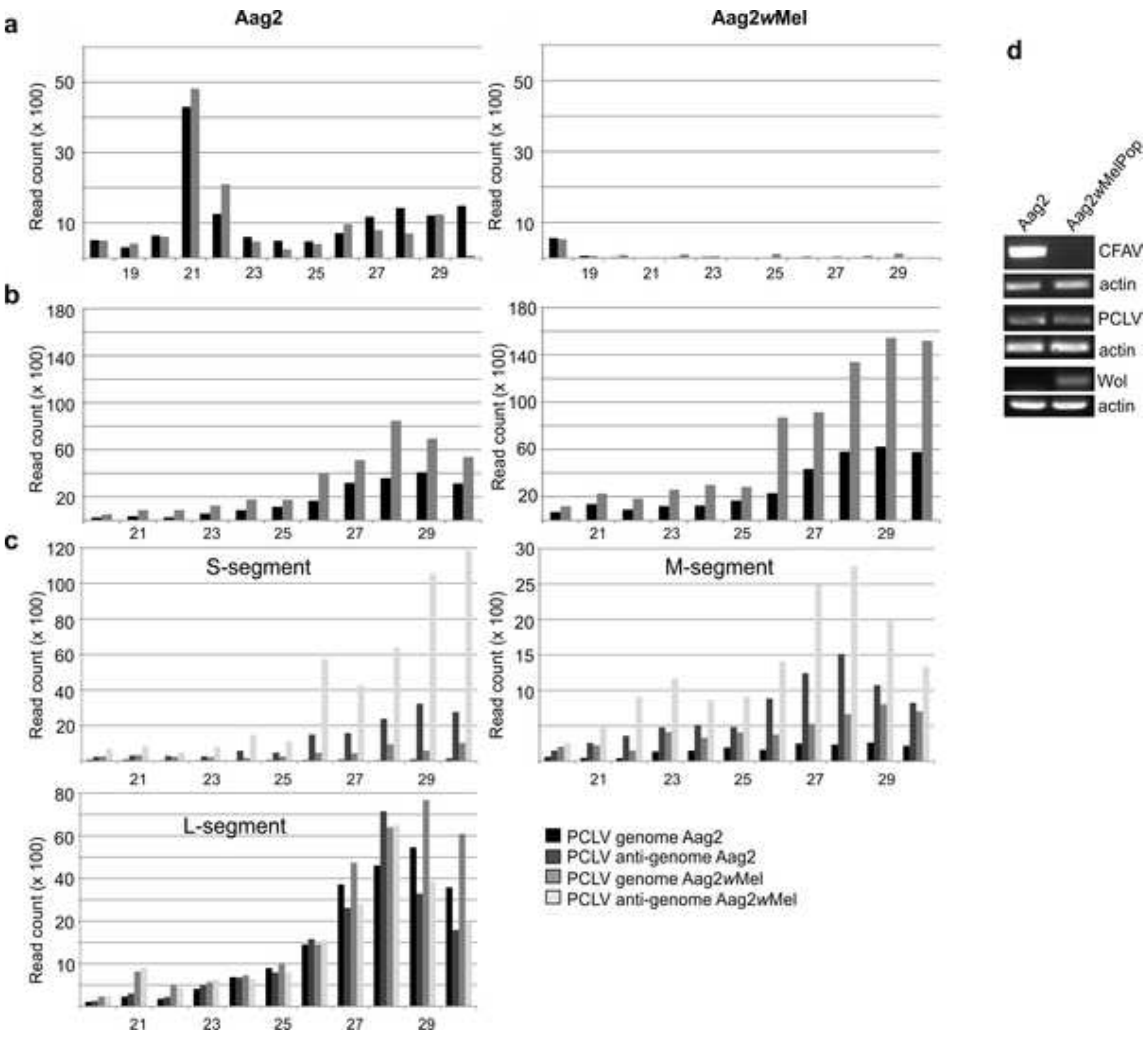

 Click here to download Figure Figure 2.tif

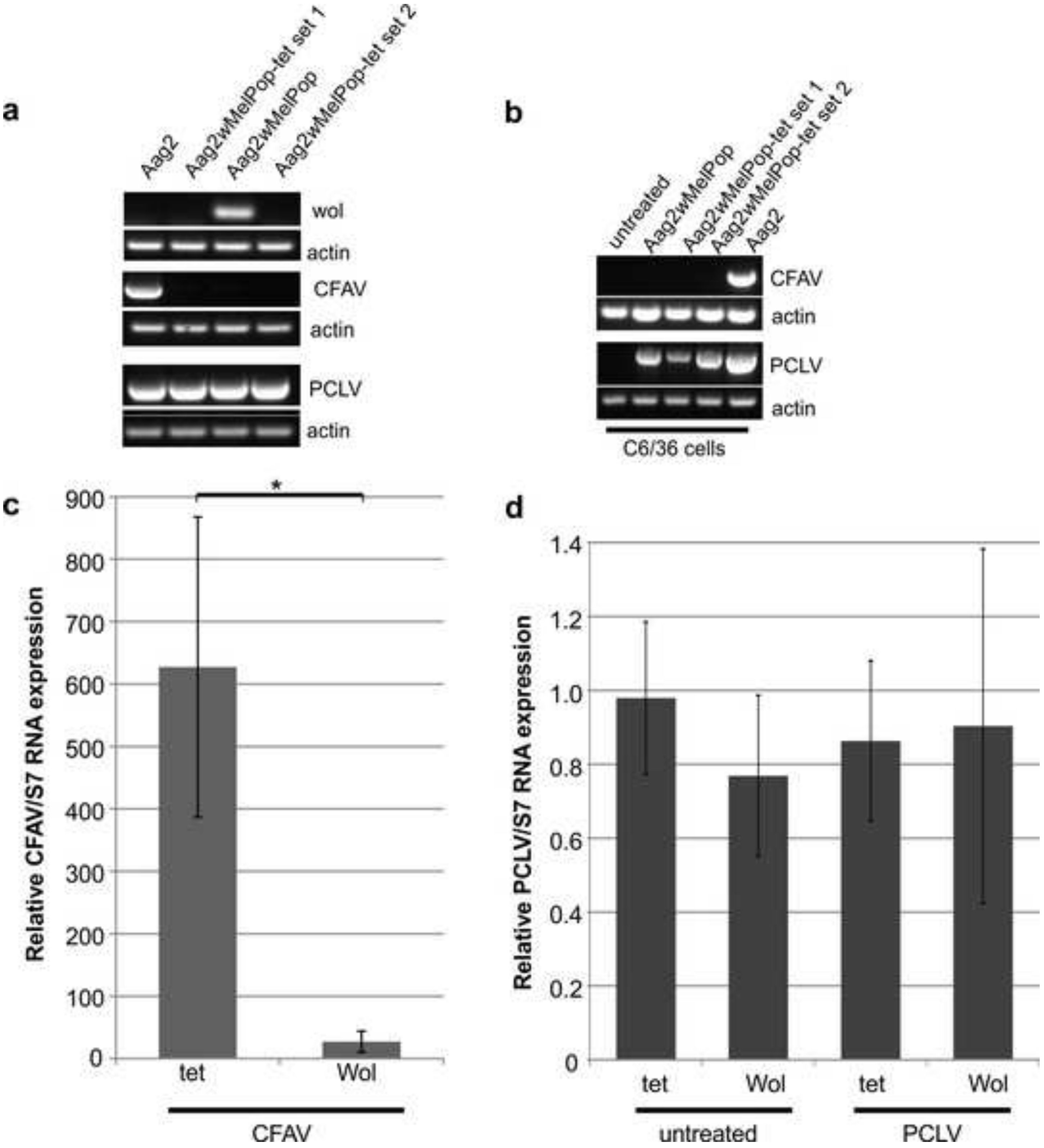

Supplementary Material Files
Click here to download Supplementary Material Files
Supplementary data Schnettler et al1.pdf

Supplementary data Schnettler et al.

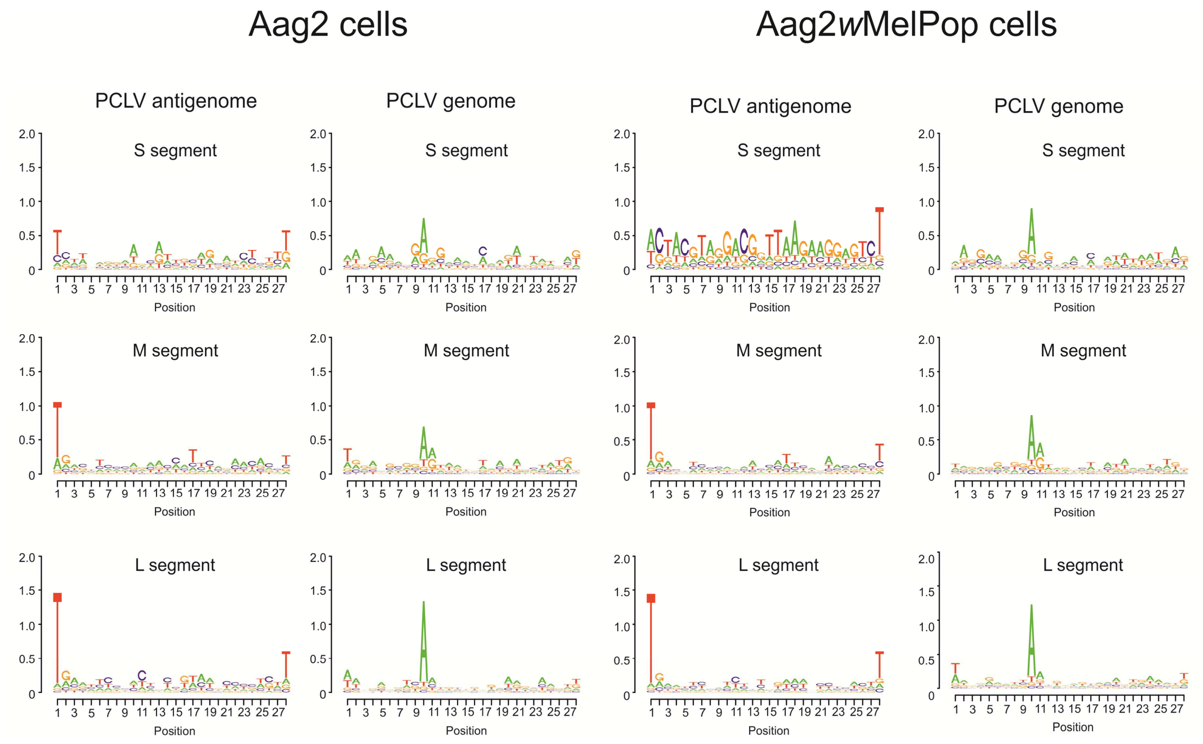

Fig. S1: PCLV specific piRNAs in Aag2 cells.

Relative nucleotide frequency and conservation per position of 28 nt long small RNAs mapping to the genome and antigenome of PCLV in Aag2 and Aag2*w*MelPop cells are indicated. Sequence is represented as DNA.

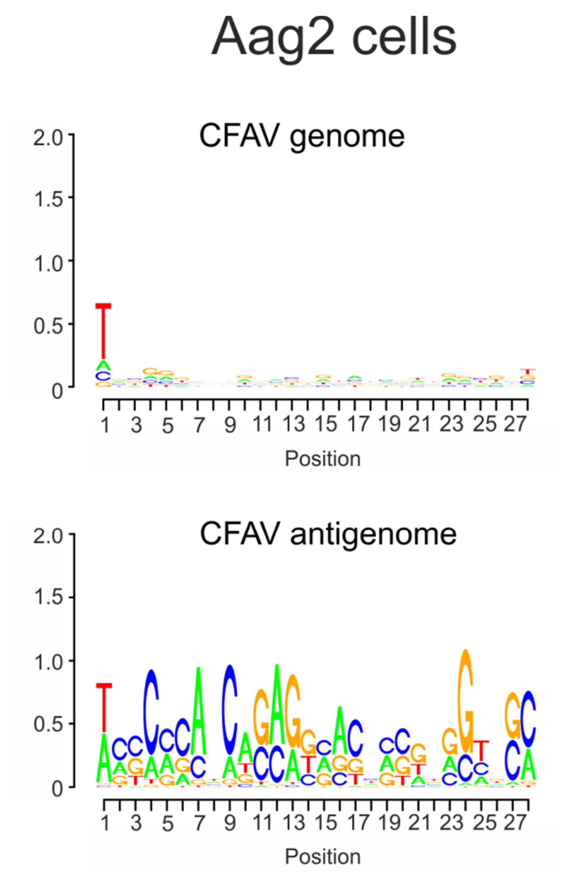

Fig. S2: CFAV specific piRNAs in Aag2 cells.

Relative nucleotide frequency and conservation per position of 28 nt small RNAs mapping to the genome and antigenome of CFAV in Aag2 cells are indicated. Sequence is represented as DNA.

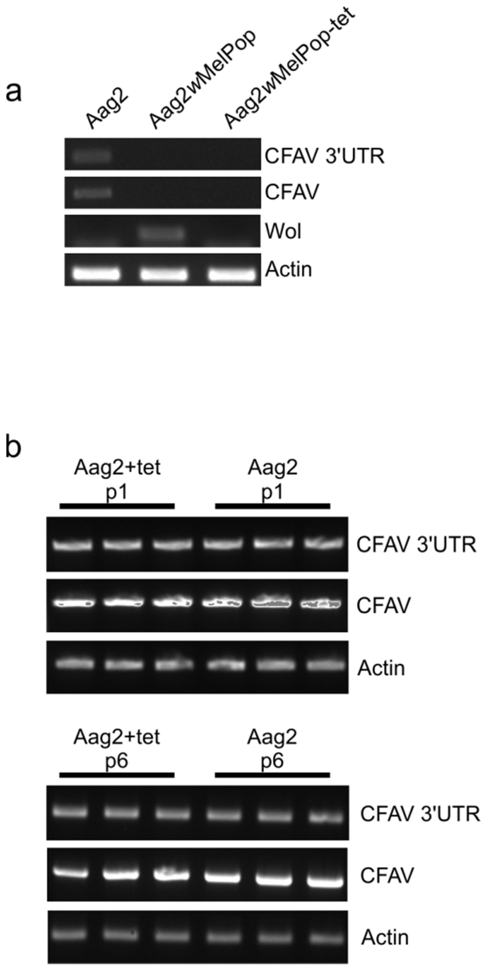

Fig. S3: Effect of tetracycline treatment on CFAV infection in Aag2 cells. (a) Detection of CFAV (two different primer pairs: 3`UTR or non-structural region) and *Wolbachia* in Aag2, Aag2*w*MelPop and Aag2*w*MelPop cells treated with tetracycline by RT-PCR. Actin was used as loading control. **(b)** Detection of CFAV (two different primer pairs; 3`UTR or non-structural region) in Aag2 or Aag2 cells treated with tetracycline cells at different time points (passage 1 top or passage 6 bottom) by RT-PCR. Actin was used as loading control.
